# Nasal shedding of bovine coronavirus during commingling is associated with reduced average daily gain and lung lesions in weaned seropositive calves

**DOI:** 10.3168/jds.2026-28706

**Published:** 2026-10

**Authors:** Joanna Urbaniec, Alkiviadis Anagnostopoulos, Peers Davies, Federico G. Hoffmann, Noelle Noyes, Joseph Neary

**Affiliations:** 1 Institute of Infection, Veterinary and Ecological Sciences, University of Liverpool, Liverpool L69 7ZB, United Kingdom; 2 Department of Biochemistry, Nutrition and Health Promotion, Mississippi State University, Mississippi State, MS 39762; 3 Institute for Genomics, Biocomputing and Biotechnology, Mississippi State University, Mississippi State, MS 39762; 4 College of Veterinary Medicine, University of Minnesota, St. Paul, MN 55108

**Keywords:** calf pneumonia, viral transmission, stress-response, bovine coronavirus, phylogenetics

## Abstract

Bovine coronavirus (BCV) is a common calf pathogen and has been proposed as a potential contributor to bovine respiratory disease (BRD); however, little is known about its epidemiology and genetic diversity on British farms. The aim of this study was to model the relationship between mixing duration and intensity, BCV shedding, BRD development, and production outcomes during a controlled commingling event designed to mimic conventional farming conditions. Three cohorts of weaned Angus × Holstein calves (8–10 wk old; n = 120) sourced from 10 British source farms (SF) were enrolled and quasirandomly assigned to mixing groups of varying intensity. Mixing intensity (MI) was defined by richness, (i.e., the number of SF contributing to each pen and evenness—the relative proportion of calves from each SF). Mixing intensity ranged from low richness and low evenness (all calves in a pen originating from a single SF) to high richness and high evenness (2 calves from 5 different SF in a single pen). Nasal swabs and fecal samples were collected on source farms on animal collection day (SF collection), d 0, 3, 7 and 21 after mixing, and analyzed by BCV quantitative PCR and Sanger sequencing. Serum samples were taken upon SF collection, d 0 and 21 postmixing, and tested for BCV-specific IgG using ELISA. Calves were monitored daily for signs clinical respiratory disease using a modified University of Wisconsin–Madison system, and presence of lung lesions was assessed by thoracic ultrasonography on SF collection and d 0 and 21 after mixing. Calves were weighed on SF, and at the beginning and end of mixing to determine ADG. Associations between MI, BCV shedding, clinical respiratory disease, lung lesions, and ADG were investigated using mixed-effect models appropriate to the outcome type, with calf defined as the experimental unit. High serum BCV IgG titers were detected in all calves at enrollment and declined steadily over the study period. Seropositivity did not prevent BCV shedding, and overall genetic diversity among isolates was low (∼2%). However, distinct genetic variants were identified between source farms and, in one case, within the same animal at the beginning and end of the study period. Nasal BCV shedding was not associated with clinical disease within 7 d of recorded symptoms. Conversely, high shedders were significantly more likely (∼4.5 times; 95% CI: 1.21, 6.8) to have concurrent lung lesions and grew slower (−0.38 kg/d; 95% CI: −0.62, −0.14) when compared with nonshedders. Concurrently, the odds of clinical BRD increased with both commingling duration and MI; however, clinical BRD was not meaningfully associated with ADG. Overall, these findings suggest that in BCV seropositive commingled calves, BCV infection is widespread but largely subclinical; importantly, high levels of virus shedding are associated with measurably worse production outcomes whereas BRD risk is primarily influenced by the duration and intensity of mixing.

## Abbreviations

The list of standard abbreviations for JDS is available at adsa.org/jds-abbreviations-24. Nonstandard abbreviations are available in the Notes.

## INTRODUCTION

Bovine respiratory disease (**BRD**) is a complex multifactorial disease, often caused by co-infection or sequential infections of both bacterial and viral pathogens. Clinical signs of BRD include fever, eye or nasal discharge, cough, loss of appetite, and in more severe cases significant respiratory distress and death (Gaudino et al., 2022). However, a considerable portion of cases are subclinical, manifesting only as pathological lung changes and reduced weight gain, thereby making diagnosis and on-farm management of BRD a significant challenge. A recent study identified that subclinical pneumonia is highly prevalent in United Kingdom (**UK**) dairy calves, affecting up to 29% of animals at peak prevalence, whereas clinical signs frequently fail to identify these cases (Lindley et al., 2025). Furthermore, it also consistently ranks as one of the top causes of morbidity and mortality in calves (Short and Lombard, 2020).

Bovine coronavirus (**BCV**) is one of the most common viral pathogens identified in BRD diagnostic submissions (Pardon et al., 2020; Buczinski, Broes and Savard, 2024). However, ambiguity remains regarding its capability to cause clinical disease as the primary agent. Although several BCV challenge studies reported clinical signs (Saif et al., 1986; Cho et al., 2001), others reported no signs of disease (Kapil et al., 1991; Park et al., 2007). Furthermore, aforementioned challenge studies were performed on newborn, colostrum-deprived animals, which are inherently at a greater risk of infection with clinical consequences due to absence of circulating antibodies (Chase et al., 2008), which might not be representative of conventional farming conditions. Under those conditions, a major risk factor for BRD are commingling events (mixing of previously unfamiliar calves). Commingling often involves transport, for example to an auction market or a feedlot; hence why BRD is also referred to as “shipping fever.” In this context, experimental evidence suggests that BCV vaccination reduces the likelihood of BRD treatment (Plummer et al., 2004), and that BCV shedding is positively associated with BRD incidence (Workman et al., 2017).

Despite this, there is limited information on circulating genetic variants of BCV and their virulence capabilities. Over 70% of publicly available sequences originate from the United States or China, with none currently available from the UK (NCBI, 2026). Improving the understanding of BCV epidemiology and genetics is therefore essential for better on-farm BRD management. Consequently, this study aimed to investigate BCV transmission, genetic diversity, and pathogenicity during a controlled commingling event in a group of weaned calves raised under standard farming practices.

## MATERIALS AND METHODS

### Study Design and Sample Size

The study was conducted on weaned Angus × Holstein calves. Ethical approval was obtained for all procedures (ASPA project PP6326528). Three temporal cohorts (n = 40 animals per cohort) were evaluated. For each cohort, calves were sourced from 5 different dairy farms. Calves were between 8 and 10 wk of age on SF collection day, and had been gradually weaned with weaning completed by 8 wk of age according to their respective SF protocols. Due to some farms supplying multiple cohorts, a total of 10 unique source farms (**SF**) were used across the entire study (see Supplemental Table S1 [see Notes] for detailed sourcing structure). Upon enrollment (1–7 d of age), a serum sample was collected from each animal and evaluated for total protein (**TP**) concentration with a Brix refractometer.

An a priori formal power calculation was not performed for this study. Estimating the expected variance required for a reliable power analysis was not feasible due to the absence of published baseline data regarding the genetic diversity of BCV strains circulating on UK dairy farms, as well as their specific effect sizes on transmission dynamics and clinical disease during commingling. Therefore, this study was designed as an exploratory investigation. The sample size (n = 120 calves across 3 temporal cohorts) was maximized based on the logistical and housing capacity of the available facilities (15 m^2^ per pen). This ensured the accurate simulation of commercial practices while strictly adhering to UK animal welfare regulations regarding maximum stocking densities (minimum 1.5 m^2^ per calf; Defra, 2007). Furthermore, this sample size is comparable to those used in previously published experimental viral transmission and coronavirus challenge trials in calves (Saif et al., 1986; Kapil et al., 1991; Cho et al., 2001; Park et al., 2007).

Details of calf transport, including vehicle disinfection, are described in detail elsewhere (Urbaniec et al.*,* 2025). Upon arrival at the research facility, calves underwent a 12- to 14-d quarantine period, maintained within their original SF groups in solid-sided pens (Buitelaar High Health Units). Quarantine was implemented to allow animals to acclimatize to the new diet and housing environment before study commencement. This was pertinent to concurrent microbiome sampling associated with a larger-scale study on BRD epidemiology. Calves were housed in the same facility throughout the entire study, and moved to their respective mixing pens on d 0 of the mixing period, with the exception of the homogeneous (control) group, which remained in the same pen throughout. Calves had no opportunity for direct contact between groups; however, pens were located outdoors, and therefore a possibility of between-pen aerosol exposure could not be definitely excluded; pen air sampling results are described further in Urbaniec et al. (2025).

Pens were bedded with straw which was supplemented daily without entering the pen. Calves were fed TMR ad libitum, prepared on-site throughout the entire study period. Pens were located in an outdoor facility, facing westerly, with airflow driven by environmental conditions. Each pen was 5 m deep and 3 m wide with smooth, solid double-walled plastic partitions from floor to ceiling. Pens featured a single-span galvanized gate at the front (2.5 m height) and a solid back wall (1.22 m height) with a 1.0 m opening above it to the roof. An insulated box-profile roof spanned 3 pens within one unit, with a 0.5-m gap between units. Pen layout was identical, featuring a 30-L wall-mounted water trough (DBL2, JFC Agri) and gate-mounted feed troughs (Gate Feeder, JFC Agri).

Following quarantine, calves from each cohort were assigned into 4 mixing pens of 10 calves each. Due to practical constraints assignment was partially randomized (i.e., based on the number of calves enrolled from each SF). Within SF, calves were randomly assigned to 2 pen types; for instance, a SF providing 12 calves would have 10 calves assigned to the homogeneous (control) pen and 2 calves assigned to the high-mixing pen (see Supplemental Table S1 and Figure 1 for detailed structure). Assignment was further stratified within the strata of sex, so that 5 male and 5 female calves were assigned to each pen type.

**Figure 1:**
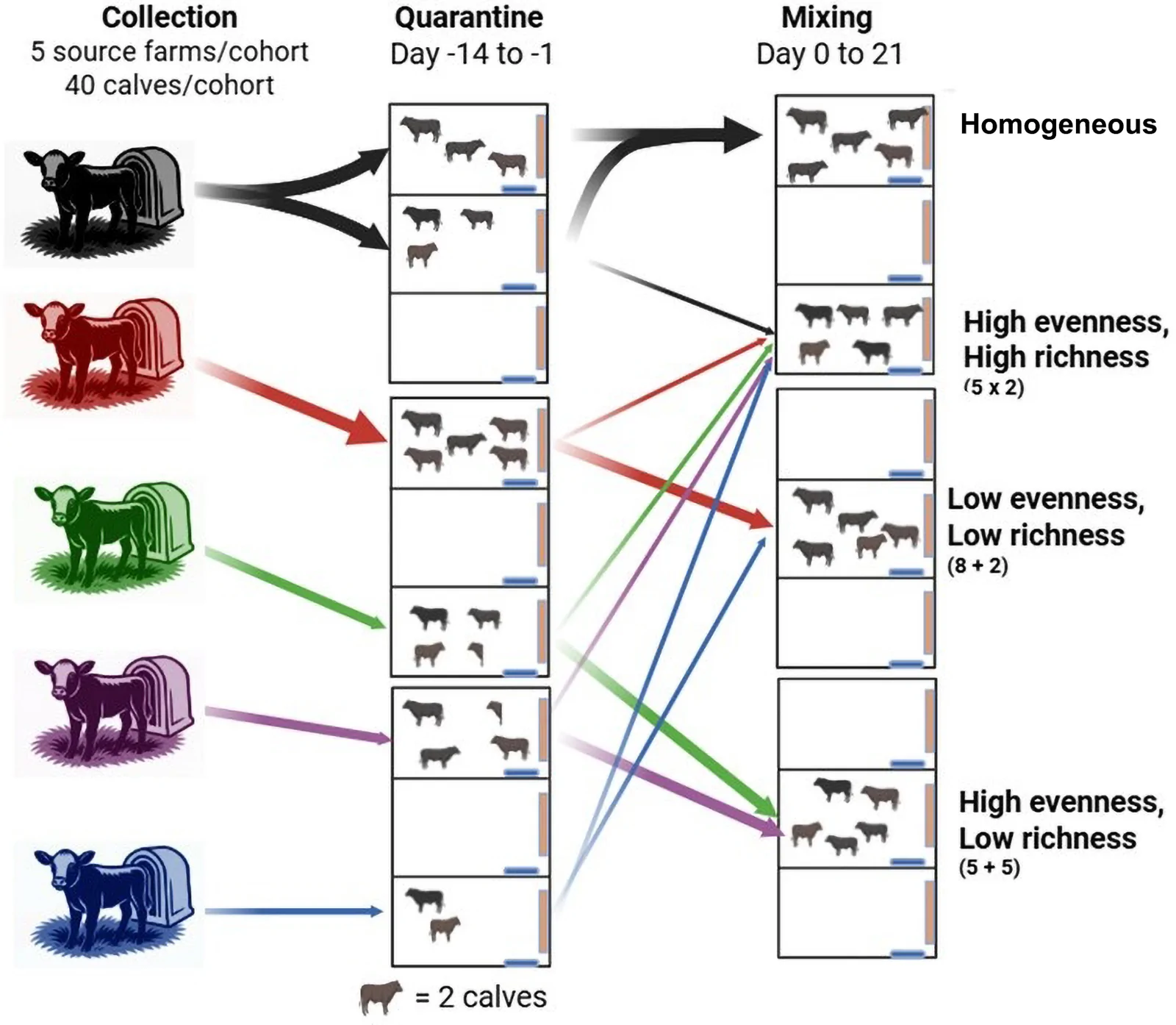
Study setup. Pen order was randomized between cohorts. Richness refers to number of different source farms in the pen. Evenness refers to relative proportion of calves from those farms in the pen. Created in https://BioRender.com.

The mixing treatments varied by richness (R; the number of different source farms in a pen) and evenness (E; the relative proportion of calves from those farms). The 4 treatment groups were

1. Homogeneous: single-source pen (low R and high E),
2. Low E and low R: 2 calves from 1 SF and 8 calves from a second SF,
3. High E and low R: 5 calves from 1 SF and 5 calves from a second SF, and
4. High E and high R: 2 calves from each of the 5 cohort SF.

Pen order was randomized between groups to minimize potential pen location effects. Calves remained in their respective commingling groups for 21 d. Observers were not blinded to pen allocation.

Meteorological data, including air temperature, rainfall, and wind speed, were recorded daily from the Met Office Synoptic Automatic Weather Station at Hawarden Airport (located 10.3 km south from the research facility) to monitor environmental conditions throughout the 3 study periods. Seasonal variation was observed between cohorts (Supplemental Table S4, see Notes), with cohort 3 experiencing notably colder temperatures (mean 6.1°C ± 2.9°C) compared with cohorts 1 and 2 (means 14.5°C ± 2.2°C and 13.3°C ± 2.5°C, respectively); these differences were accounted for by including cohort as a forced fixed effect in all statistical models.

### Sample Collection

All samples were collected starting with the homogeneous (control) pen.

Deep nasal (**NL**) swabs and fecal samples were collected from each calf at the SF on the day of collection, and subsequently on d 0, 3, 7, and 21, relative to mixing. The NL samples were collected using an autoclaved 8-inch (20.3-cm) Jumbo Rayon-tipped swabs (Medline). The swab was inserted into the left nostril at approximately three-quarters of the shaft length and twisted approximately one-quarter turn 3 times before being removed. The tip was snapped off into 3 mL of DNA/RNA shield (Zymo Research) and stored at −20°C until further processing. A sterile swab exposed to the pen environment for approximately 1 min served as a negative environmental control.

Fecal samples were collected directly from the rectum into sterile pots and stored at −20°C until further processing. Blood samples were collected via jugular venipuncture into plain red-top tubes (BD Vacutainer). Samples were allowed to clot at room temperature for 2 to 4 h or refrigerated overnight. Following clotting, samples were centrifuged at 1,000 × *g* for 10 min at room temperature. Serum was aliquoted into sterile microcentrifuge tubes and stored at −20°C until further analysis.

### Thoracic Ultrasonography

The presence of pathological lung changes was assessed by a trained veterinarian (AA/JN) using thoracic ultrasonography (**TUS**) on the source farm collection day, the day of mixing, and at the end of the mixing period (d 21 after mixing), as described previously (Ollivett and Buczinski, 2016).

Briefly, calves were restrained in a crush with a head restraint. The scanning area was wiped with 70% ethanol, and ultrasound coupling gel (CVET, Covetrus) was applied. The thorax was scanned with a linear probe (Draminski 4VET Mini, Draminski Technology) set at a frequency of 5 MHz (6–9 cm depth). Scanning proceeded in a caudal-to-cranial direction on both sides of the thorax, evaluating the tenth through the first intercostal spaces by moving the probe parallel to the ribs.

Lung consolidation was classified using a 5-point progressive scoring system (Table 1). Animals with a TUS score of 2 or higher (lobular lesions ≥1 cm) were classified as having lung lesions (**LL**; indicative of subclinical pneumonia in the absence of clinical signs).

**Table 1:** Thoracic ultrasonography scoring system^1^

TUS score	Definition
0	Healthy lung
1	Lobular lesions <1 cm
2	Lobular lesions ≥1 cm
3	Lobar lesion (full-thickness consolidation of a single lobe)
4	Lobar lesions (full-thickness consolidation of a multiple lobes)

1 Reproduced from Ollivett and Buczinski (2016) under CC-BY 4.0 license. Modified by merging TUS scores 4 and 5 into a single category (score 4).

### Clinical Pneumonia Evaluation

Calves were evaluated once daily for signs of clinical pneumonia using a modified University of Wisconsin–Madison scoring system (Table 2). To minimize the risk of fomite transmission between mixing groups, evaluations were performed visually from outside the pen during feeding times. Consequently, rectal temperature and induced cough were excluded from the scoring. Calves with a cumulative score of 4 or more points were considered to have clinical pneumonia. Scoring was performed predominantly by a single experienced observer (GC), with a minority of daily scores (<10%) recorded by other trained observers (JN or JU) due to logistical reasons.

**Table 2:** Clinical pneumonia scoring system

Point	Eyes	Nose	Ears	Cough
0	No discharge	No discharge	Normal	None
1	Small amount of discharge	Unilateral cloudy discharge	Ear flick or head shake	NA^1^
2	Moderate amount bilateral discharge	Bilateral cloudy discharge or excessive mucus	Unilateral droop	Occasional spontaneous cough
3	Heavy ocular discharge	Copious bilateral discharge; mucopurulent	Head tilt or bilateral droop	Repeated spontaneous cough

1 NA = induced cough was not assessed to minimize pen entry requirements.

### ADG Estimation

Calves were weighed using a Tru-Test aluminum weigh platforms (1.1 or 2.2 m) and Tru-Test S3 weigh scale with MP600 load bars (Datamars UK), with a precision of ± 0.5 kg. Calves were weighed 3 times: on source farm collection day, d 1 after mixing, and at the end of the mixing period (d 21 after mixing). Average daily gain (kg/d) during quarantine and mixing periods was calculated by subtracting the initial weight from the final weight and dividing by the number of days between measurements (20 d for mixing period, and 14–11 d for quarantine period).

### Bovine Coronavirus IgG ELISA

Anti-BCV antibodies present in serum samples were captured with a commercially available ELISA assay (SVANOVIR BCV-Ab; Boehringer Ingelheim Svanova, Uppsala, Sweden). Assay was performed following manufacturer's instructions and quality control cutoffs.

Briefly, serum samples were thawed at 4°C overnight, diluted 1:50 in sample dilution buffer and 100 µL/well was added to a plate precoated with either BCV antigen or the control antigen. Positive control and negative control serums provided in the kit were also run in duplicate on each plate. Plates were statically incubated at 37°C for 1 h and washed 3 times with PBS-Tween20 (0.1%) solution. 100 µL/well of secondary HRP-conjugated (anti-bovine IgG_1_) antibody was then added to the plate. Plates were again incubated for 1 h at 37°C and washed 3 times with PBS-Tween20 solution; 50 µL/well of HRP substrate solution was added and the plate, and the plate was incubated for 10 min at room temperature in the dark. Afterward, the reaction was stopped by adding 50 µL/well of stop solution. Absorbance at 450 nm was read immediately using a ClarioStar spectrophotometer (BMG Labtech). Optical density (**OD**) values for BCV antigen were corrected for OD of corresponding control antigen for each sample (**ODCorr**).

The percentage positive value (**%PP**) was calculated using the following formula:

%PP = ODCorr(Sample or Negative Control)/ODCorr(Positive Control) × 100%.

Each sample was run in duplicate and an any samples with >25% discrepancy in results between replicates were rerun.

Source farm dry-cow vaccination protocols were evaluated to assess their effect on arrival BCV antibody levels. Farms were categorized based on the BCV-containing vaccine brand used: RotaVec Corona (MSD Animal Health; 6 farms), other vaccine brands (Bovigen, Virbac, or Fencovis, Boehringer Ingelheim; 3 farms), or unvaccinated (one farm).

### RNA Extraction and Quantitative PCR

Total RNA from NL swabs was extracted with Quick-DNA/RNA Miniprep kit (Zymo Research). One hundred milligrams of swab material was cut from the tip using sterile scissors. The swab was first homogenized using TissueLyzer II (Qiagen, Hilden, Germany): 400 μL of kit lysis buffer was added to the weighed-out swab material in a sterile Eppendorf tube containing 1 autoclaved 5-mm stainless steel ball (Sourcing MAP*)* and shaken twice for 3 min at 2.5 Hz. Afterward, the sample was centrifuged at 10,000 × *g* for 1 min at room temperature, the liquid was transferred to the extraction column and extraction proceeded according to manufacturer's protocol. The RNA was eluted in 50 µL of RNase-free water. Homogenized mock (sterile swab) sample, spiked with BCV M gene RNA standard (bp 28,841–29,298) was used to determine limit of detection (**LOD**) of the assay, as previously described (Decaro et al., 2008).

Total RNA from fecal samples was extracted using Quick-RNA Viral kit (Zymo Research), according to manufacturer's instructions. Samples were preprocessed with the BioPrep feces preparation kit (BioSellal) according to manufacturer's instructions, with the exception of using viral RNA buffer (Zymo Research), rather than supplied lysis buffer. Two grams of fecal material was processed per extraction, and RNA was eluted in 50 µL of RNase-free water. Mock sample (sterile water) spiked with BCV M gene RNA standard was uses to determine the LOD of the assay.

The BCV quantitative PCR (**qPCR**) primers and probe sequences had previously been validated (Decaro et al., 2008), and bovine 18S RNA gene was used as the internal qPCR positive control (Supplemental Table S2, see Notes). All primers and probes were synthesized by integrated DNA technologies. Samples were amplified using Luna Universal Probe One-Step RT-qPCR Kit (New England Biolabs), according to manufacturer's instructions. An annealing step (15 s at 50°C) was added to the standard protocol as it was found to increase LOD, and amplification was run for 35 cycles. Reactions were carried out on the Roche LC480 instrument (Roche Diagnostics). A 10-fold increase in fluorescence relative to baseline was used as positivity cut-off and amplification curves were visually inspected to confirm amplification.

### BCV Spike Gene Hypervariable Region Sequencing

Hypervariable region of the spike protein (S) gene (bp 1,368–1,776) was PCR-amplified using previously published primers (S5F + S5R; Takiuchi et al., 2008; Supplemental Table S2). The PCR reaction was performed for 35 cycles in a standard thermocycler, using Luna one-step RT-PCR system (New England Biolabs) annealing step of 15 s at 50°C, and 50-µL reaction volume. Five microliter of each amplicon was loaded onto 1% agarose gel and electrophoresed at 100 V for 1 h. A band of 650 bp visible on the gel was used to confirm successful amplification. Successful amplicons were sent off for Sanger sequencing (GeneWiz UK) and resulting sequence traces were visually inspected for quality control.

### Phylogenetic Analysis

Phylogenetic trees were constructed in Molecular Evolutionary Genetics Analysis (**MEGA**) software v.12 (https://www.megasoftware.net/), using the maximum likelihood method and 1,000 bootstrap replicates. Before tree construction, sequences were aligned using the MUSCLE algorithm in MEGA software.

Genetic diversity metrics (average pairwise distances, π, Fu and Li's statistics) were calculated in DNASP software v.6 (University of Barcelona).

Bayesian tip-association significance testing (**BaTS**) was performed in BaTS software v.1 (Angelini et al., 2008). First, Bayesian phylogenetic inference was performed in MrBayes software v 3.2 (Huelsenbeck and Ronquist, 2001). Two independent Markov Chain Monte Carlo runs were performed, each with 4 chains (1 cold and 3 heated). Runs continued for 5 million generations with sampling every 1,000 generations. Convergence was assessed using the average standard deviation of split frequencies (**ASDSF**), effective sample sizes (**ESS**), and potential scale reduction factors (**PSRF**). Adequate convergence was assumed with ASDSF ≈0.01, PSRF ≈1, and ESS >800. The first 25% of samples were discarded as burn-in. Posterior trees from both independent runs were combined and used for phylogenetic traits association in BaTS. Statistical support for trait-phylogeny association was evaluated using the association index (**AI**), parsimony score (**PS**), and monophyletic clade (**MC**) statistics, with significance assessed by comparison to null distributions generated by randomization of trait states across the tree tips (1,000 randomizations per trait). Due to small and uneven sample sizes for some traits, PS and MC statistics were interpreted only for well-sampled groups (n > 10).

### Statistical Analysis

For analysis, calf was defined as the experimental unit. Pen-level BCV IgG levels over time were compared using a repeated-measures ANOVA, with pen specified as the subject and repeated measurements over time (sampling days).

A linear mixed-effects model was used to evaluate the association between BCV antibody levels at source farm collection, maternal dry-cow vaccination categories (RotaVec Corona, alternative vaccine brands, or unvaccinated), and individual calf serum TP concentrations (sampled 2–7 d after birth). Source farm was included as a random effect to account for clustering.

Logistic regression was used to assess the association between BCV antibody levels and BCV shedding status (nasal or fecal) at SF collection day, with BCV shedding as the binary outcome variable and %PP BCV IgG as the predictor.

Bovine coronavirus viral copy number differences between sampling time points or sample types (nasal/fecal) were compared using a 2-tailed Mann–Whitney U test.

The preceding analyses were performed in GraphPad Prism version 10.6.1 (GraphPad Software) or Minitab Statistical Software v.22.3.0 (Minitab). All other analyses were performed in RStudio version 4.4.1 using the *dplyr*, *lme4*, *MuMIn*, *car*, and *performance* packages. Calibration plots for logistic regression models were generated using the *ggplot2* package.

All models were constructed using stepwise regression, with variables with *P* < 0.1 retained. Adequate model fit was confirmed through visual evaluation of diagnostic plots generated using the *performance* package in R studio. Bovine coronavirus shedding (nasal and fecal), mixing treatment (pen type), and commingling length (with the exception of the ADG model) were included a priori in all models as variables of interest. Cohort was included as a control variable regardless of statistical significance, and nasal BCV shedding on d 0 was included in BCV shedding model as an additional control variable. Source farm variable was excluded from all models due to high collinearity with mixing treatment (pen type). This collinearity was unavoidable due to practical constraints of the study, as some farms were able to supply only a limited number of calves or contributed to a single study cohort.

There were no predefined exclusion criteria for enrolled animals and available data were included in the analysis included unless otherwise specified. One animal from cohort 3 was excluded from d 21 dataset due to euthanasia for compassionate reasons before this time point; the cause of death was unrelated to respiratory disease. Additionally, 9 fecal samples did not yield enough material for BCV qPCR, which is further stated in corresponding figure legends.

Across all models nasal BCV shedding variable was categorized into 3 groups: no detectable shedding, low shedding (<10^7^ copies/µL), and high shedding (≥10^7^ copies/µL), based. The cutoffs for low and high shedding were based on the histogram of positive values. Fecal shedding was a binary variable across all models due to majority of positive samples clustering around the limit of detection of the qPCR assay. Overall significance of categorical variables was assessed using type II ANOVA.

The relationship between nasal BCV shedding and commingling length (study day, categorical variable) and mixing treatment (pen type) was investigated using a generalized linear mixed model (**GLMM**) with a binomial distribution and logit link function. Calf ID was included as a random effect to account for repeated measurements. Mixing treatment, study day, fecal BCV shedding, and nasal BCV shedding on the mixing day (yes/no) were included as fixed effects. Variables of TP at birth and BCV serum IgG levels were removed during backward elimination process.

The relationship between the incidence of clinical respiratory disease during commingling (defined as animals with a clinical score ≥4), commingling length, mixing treatment, and BCV shedding was investigated using a GLMM with a binomial distribution and logit link function. Because clinical signs were recorded daily whereas other variables were measured periodically, commingling length was categorized into 3 biologically meaningful periods: early (3–6 d after mixing), mid (7–14 d after mixing), and late (15–21 d after mixing). The maximum clinical pneumonia score recorded within each period was used to represent peak disease severity for that timeframe. To account for repeated measurements across these 3 periods, calf ID was included as a random effect. Mixing treatment, study period, and BCV shedding (nasal or fecal) were included as fixed effects. A history of clinical pneumonia (defined as clinical pneumonia score ≥4 during the quarantine period, including the first 48 h after mixing) was included to account for baseline predisposition to respiratory disease. Variables of TP at birth and BCV serum IgG levels were removed during backward elimination process.

Factors associated with the development of LL (TUS ≥2) during commingling (calves with no LL on d 0 and LL on d 21) were not modeled due to the high and uneven baseline prevalence of LL (10%–70% baseline pen-level prevalence), which would prevent reliable estimation of effects, as pens with high baseline prevalence had few calves that could develop LL.

To evaluate factors associated with the concurrent presence of LL during commingling (defined as TUS score ≥2, measured on d 0 and 21 of the mixing period), a GLMM with a binomial distribution and logit link was used. Calf ID was included as a random effect to account for repeated measurements. Mixing treatment (pen type), concurrent BCV shedding (nasal and fecal), and history of LL (TUS score ≥2 at SF collection) were included as fixed effects. Total protein at birth and BCV serum IgG levels were removed during the backward elimination process.

The association between ADG (kg/d) during mixing period, BCV shedding (nasal and fecal), and respiratory disease (CPN and LL) was investigated using an ordinary least squares linear regression model. Cumulative CPN score during the mixing period and LL category based on cumulative score from d 0 and 21 of mixing were included in the model a priori as variables of interest. Fixed effects included study cohort, mixing treatment (pen type), fecal or nasal BCV shedding at any point during mixing period, and ADG during quarantine period. Lung lesions were categorized into 3 groups: healthy (cumulative score 0–2), moderate (3–4), and severe (≥5). Fecal BCV shedding at any point during mixing period and cumulative CPN and TUS scores during the quarantine period were removed during the backward elimination process.

## RESULTS

### Respiratory Disease Prevalence on SF

On the day of collection from the SF, the prevalence of clinical pneumonia (**CPN**) and LL varied across cohorts. The median prevalence of CPN was 14.3% for cohort 1 and 0% for cohorts 2 and 3. In contrast, LL were more prevalent across all cohorts, with a median prevalence of 66.0% for cohort 1, 14.3% for cohort 2, and 28.6% for cohort 3. At the time of sourcing, BCV shedding (nasal or fecal) was detected in all cohorts, with median on-farm prevalences of 8.3%, 40.0%, and 14.0% for cohorts 1, 2, and 3, respectively (Figure 2).

**Figure 2:**
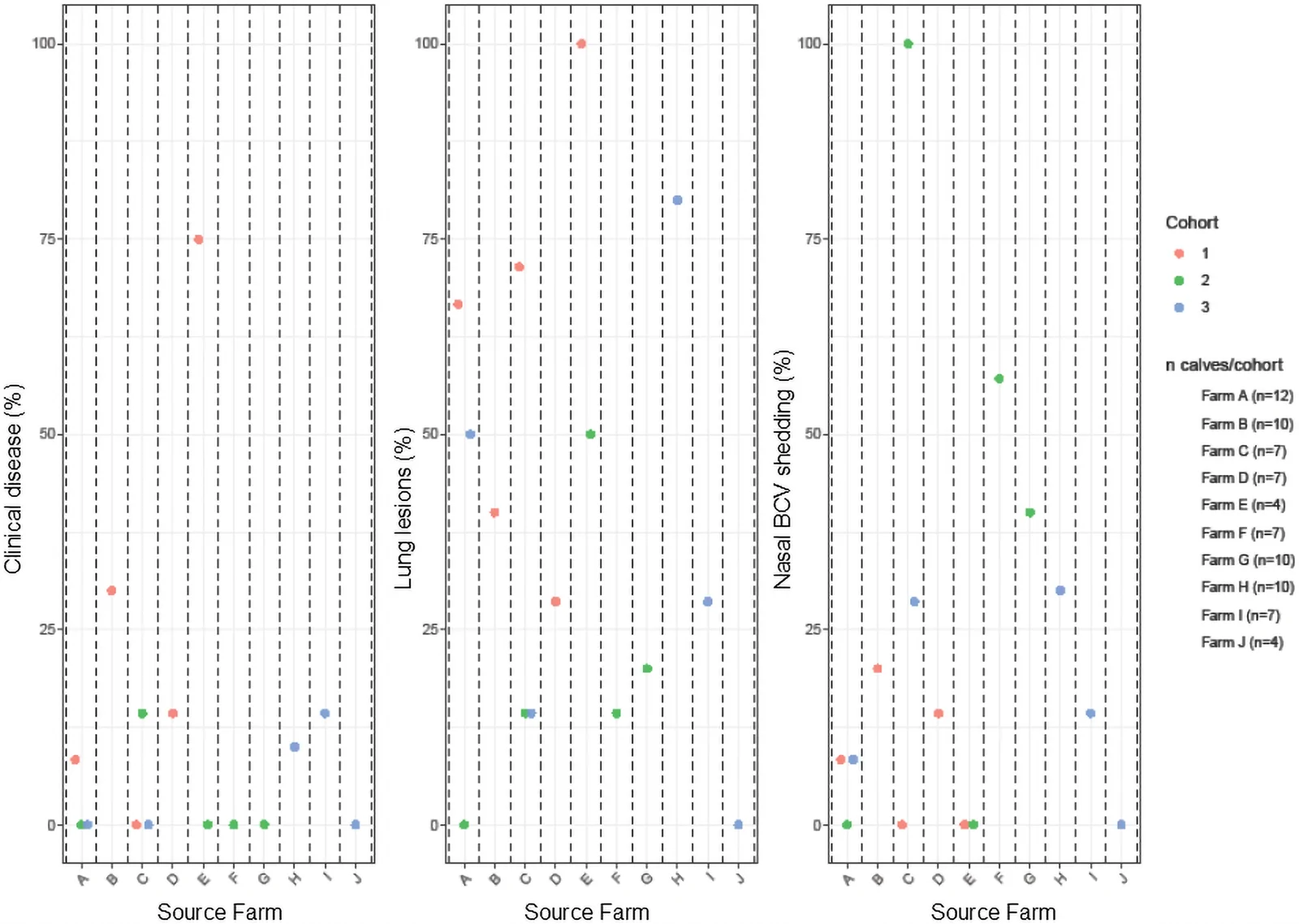
Prevalence of clinical respiratory disease, lung lesions, and bovine coronavirus (BCV) shedding in calves upon collection from source farms. Each data point represents the prevalence (%) within a single-source farm for a specific study cohort (cohort 1 = orange, cohort 2 = green, cohort 3 = blue). Clinical disease was defined as a visual respiratory score ≥4; subclinical disease was defined as a thoracic ultrasonography (TUS) score ≥2. The number of calves (n) obtained from each source farm was consistent across cohorts. Limit of detection for nasal BCV shedding was 10^4^ copies/0.1 mg swab material.

### BCV Seroprevalence

All calves enrolled onto the study (n = 120) had detectable BCV IgG_1_ in the serum upon collection from their respective SF (mean %PP = 118, range 29–170, positivity cutoff = 10). Furthermore, they all remained positive throughout the study, although the average BCV IgG levels decreased with time (mean %PP = 109.0 and %PP = 101 on mixing day and d 21 after mixing, respectively, *P* = 0.005). It should be noted that the results presented in Figure 3 are semiquantitative (i.e., a %PP change by a factor of x does not necessarily correspond to a proportional change in BCV IgG_1_ serum levels).

**Figure 3:**
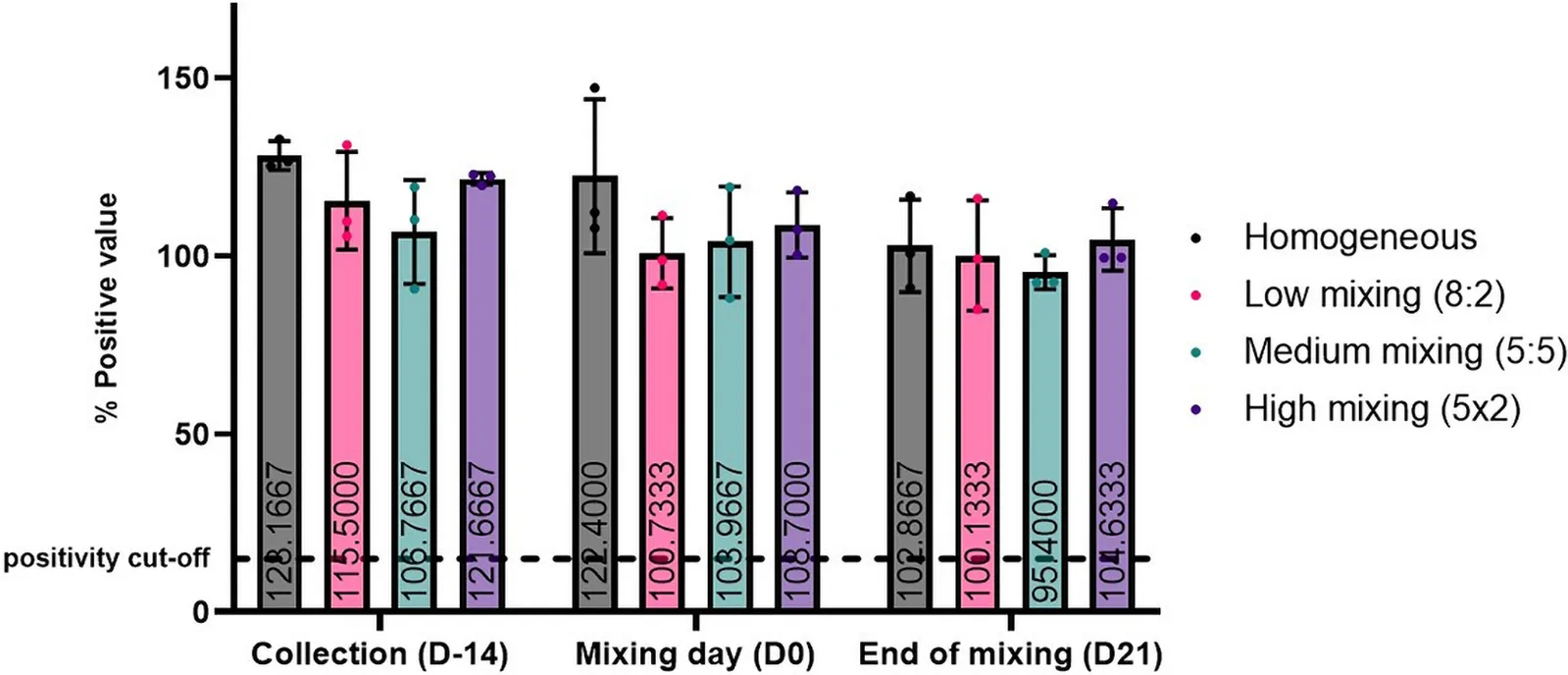
Bovine coronavirus (BCV) serum IgG_1_ levels over time, stratified by mixing treatment. Bars represent the overall mean percent positive value (%PP) for each mixing group (n = 30 calves per bar). Individual data points represent the mean of that mixing group within each of the 3 individual cohorts (n = 10 calves per dot). Error bars indicate standard deviation. The dashed line at %PP = 10 indicates the manufacturer's threshold for BCV exposure. Note that ELISA %PP values are semiquantitative; changes in %PP indicate relative reductions in antibody concentration but do not necessarily correspond to proportional changes in absolute IgG_1_ levels.

Dry-cow vaccination against neonatal enteritis pathogens was routinely administered on 9 of the 10 SF (6 used RotaVec Corona and 3 used alternative brands). Dam vaccination group had no effect on calf arrival antibody levels after accounting for individual passive transfer status (*P* = 0.19). Controlling for source farm as a random effect, each 1 g/dL increase in serum TP was associated with a 14.0%PP increase in baseline BCV antibody levels (95% CI: 8.1%–19.9%PP; *P* < 0.001). Source farm exerted a strong random effect, with an intraclass correlation coefficient of 0.37. However, higher arrival antibody levels were not associated with reduced odds of active viral shedding at source farm collection (*P* = 0.51).

### BCV Transmission During Mixing

Despite detectable serum antibodies, calves across all study cohorts shed BCV nasally and in the feces both during quarantine and mixing period (Figure 4). Overall, the median viral copy number was 10-fold higher in nasal swab samples than in fecal samples (10^5^ and 10^4^ copies/µL, respectively, *P* = 0.01). Furthermore, the median viral copy number for nasal shedding was over 100-fold higher on d 21 after mixing when compared with previous sampling days (10^6.2^ and 10^4^ copies/µL respectively, *P* < 0.001); however, fecal shedding loads remained stable over time (median of 10^4.5^ and 10^4^ copies/µL, respectively, *P* = 0.53).

**Figure 4:**
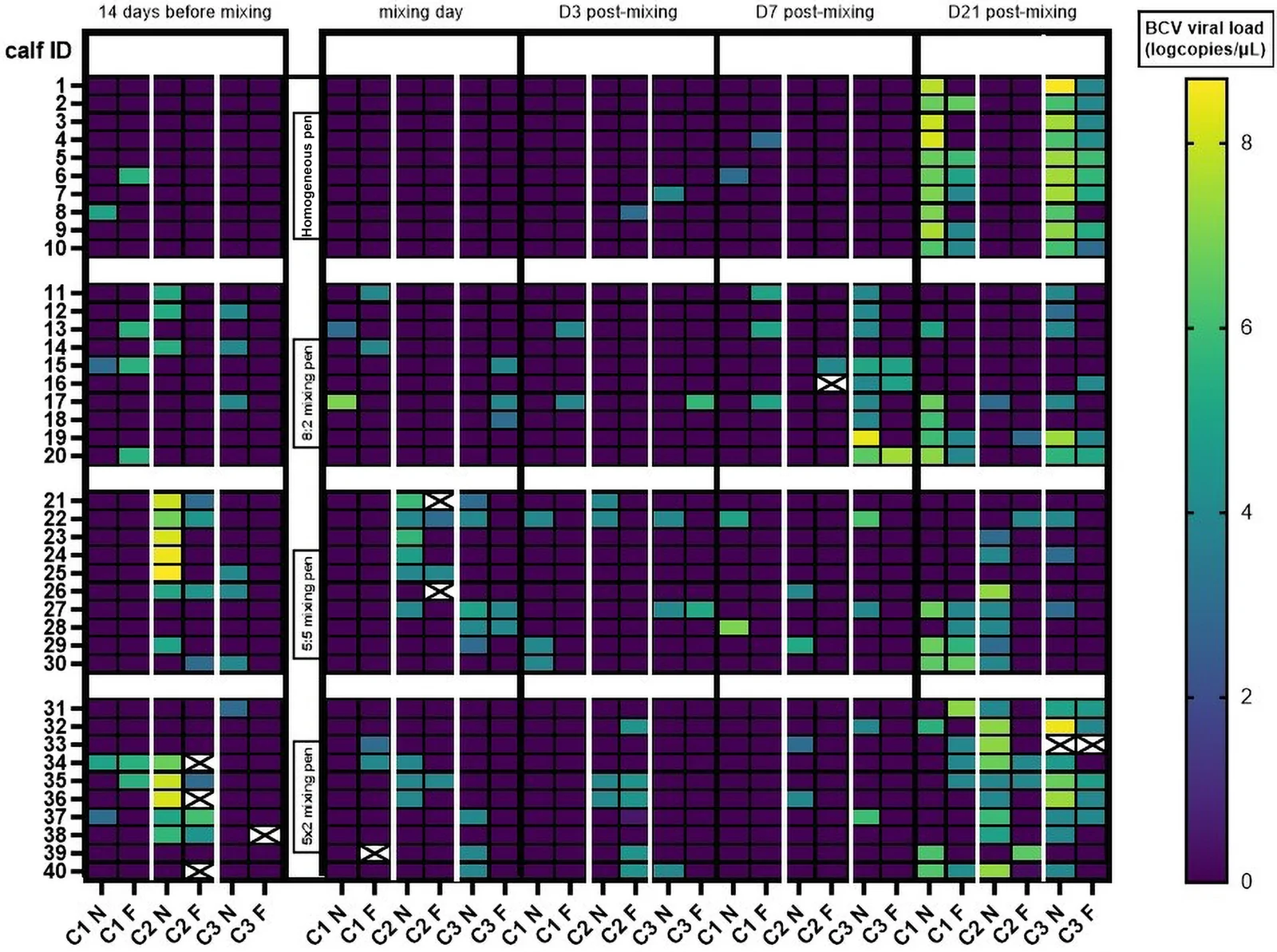
Bovine coronavirus shedding during a controlled mixing event. C = study cohort, N = nasal shedding, F = fecal shedding. Limit of detection was 10^5^ copies/g swab material (10^3^ copies/μL) and 10^6^ copies/g feces (10^4^ copies/μL). White boxes with an “X” denote missing samples due to insufficient sample material.

Variables affecting nasal BCV shedding were further evaluated with a GLMM. After a priori inclusion of variables of interest (mixing treatment [pen type] and mixing length) and forcing-in control variables (study cohort, BCV shedding baseline), only the presence of fecal BCV shedding remained as an additional fixed effect in the final model after backward stepwise elimination.

The odds of nasal shedding were mostly affected by length of mixing (*P* < 0.0001, ΔR^2^ = 0.20), study cohort (*P* = 0.008, ΔR^2^ = 0.05) and concurrent fecal shedding (*P* = 0.001, ΔR^2 =^ 0.03; Table 3). Calves were at 2.4 (95% CI: 1.02–5.69) and 16.8-fold (6.58–42.75) greater odds of shedding nasal BCV at d 7 and 21 postmixing, respectively, and at 3.9-fold (1.71–9.03) greater odds if they were shedding fecal BCV. There was no consistent effect of mixing treatment (pen type) on BCV shedding (*P* = 0.42).

**Table 3:** Factors associated with nasal bovine coronavirus (BCV) shedding during a controlled mixing event, estimated using a generalized linear mixed model with a binomial distribution and logit link; results are presented as odds ratios (OR) with 95% CI^1^

Predictor	OR	95% CI (lower; upper)	*P*-value
Cohort			
Cohort 2	1.05	0.44; 2.50	0.92
Cohort 3	3.33	1.41; 7.83	0.006**
Mixing length			
D7 after mixing	2.41	1.02; 5.69	0.04*
D21 after mixing	16.77	6.58; 42.75	<0.001***
Fecal BCV shedding	3.93	1.71; 9.03	0.001**
Mixing intensity (pen type)			
High-mixing pen	1.8	0.68; 4.82	0.24
Medium-mixing pen	1.97	0.73; 5.29	0.18
Low-mixing pen	1.02	0.37; 2.8	0.96
Nasal BCV shedding on mixing day	0.73	0.35; 1.5	0.39

1 Reference categories were cohort 1, d 3 after mixing, no fecal BCV shedding, and homogeneous (no mixing) pen.

* *P* < 0.05,

** *P* < 0.01,

*** *P* < 0.001.

### BCV Genetic Diversity

The hypervariable region (**HVR**) of the spike (S) gene was sequenced in 95 isolates across 3 study cohorts (C; 31 from C1, 33 from C2, and 31 from C3; GenBank Accession No: PZ669943-PZ670037). In the 408-bp region, 17 nonsynonymous and 20 synonymous substitution sites were identified, yielding a total of 39 distinct mutations, and 20 unique haplotypes. Overall, the average pairwise distance (**p-distance**) between isolates was ∼2%. Intragroup diversity was lowest in C2 (average p-distance ∼0.3%) and similar in C1 and C3 (∼2%). Intergroup diversity was highest between C3 and C1/C2 (average p-distance of ∼3% and ∼2.7%, respectively), and lower between C1 and C2 (∼1.38%). There was no strong evidence of purifying selection or recent population bottlenecks (Fu and Li's *F* = −0.22 [*P* > 0.1], Fu's F: 0.587 [not significant; **NS**], Tajima's D = 0.344 [NS]).

Across cohorts, sequenceable BCV was detected before mixing with varying prevalence and genetic diversity. In C1, calves from farms A (1/12) and B (3/7) were shedding BCV during the quarantine period, with farm B isolates p-distances ranging from 0.25% to 4.2% and an average between-SF p-distance of ∼2.5%. In C2, calves from farms C (7/7), G (4/10), and F (3/7) were shedding sequenceable nasal BCV; isolates within each source farm had identical HVR sequences, and between-farm average p-distances ranged from 0.1% (farms C and F) to ∼0.5% (farms C and G, and farms G and F). In C3, calves from farms C (4/7), H (1/10), and J (2/4) were shedding sequenceable nasal BCV, with isolates within SF again having an identical HVR but differing between farms on average by 2.5% to 3.6%. Finally, the average p-distance between-farm C isolates recovered in C2 and C3 was ∼0.7%.

Following mixing, isolates collapsed into clusters spanning multiple farms and pens (Figure 5).

**Figure 5:**
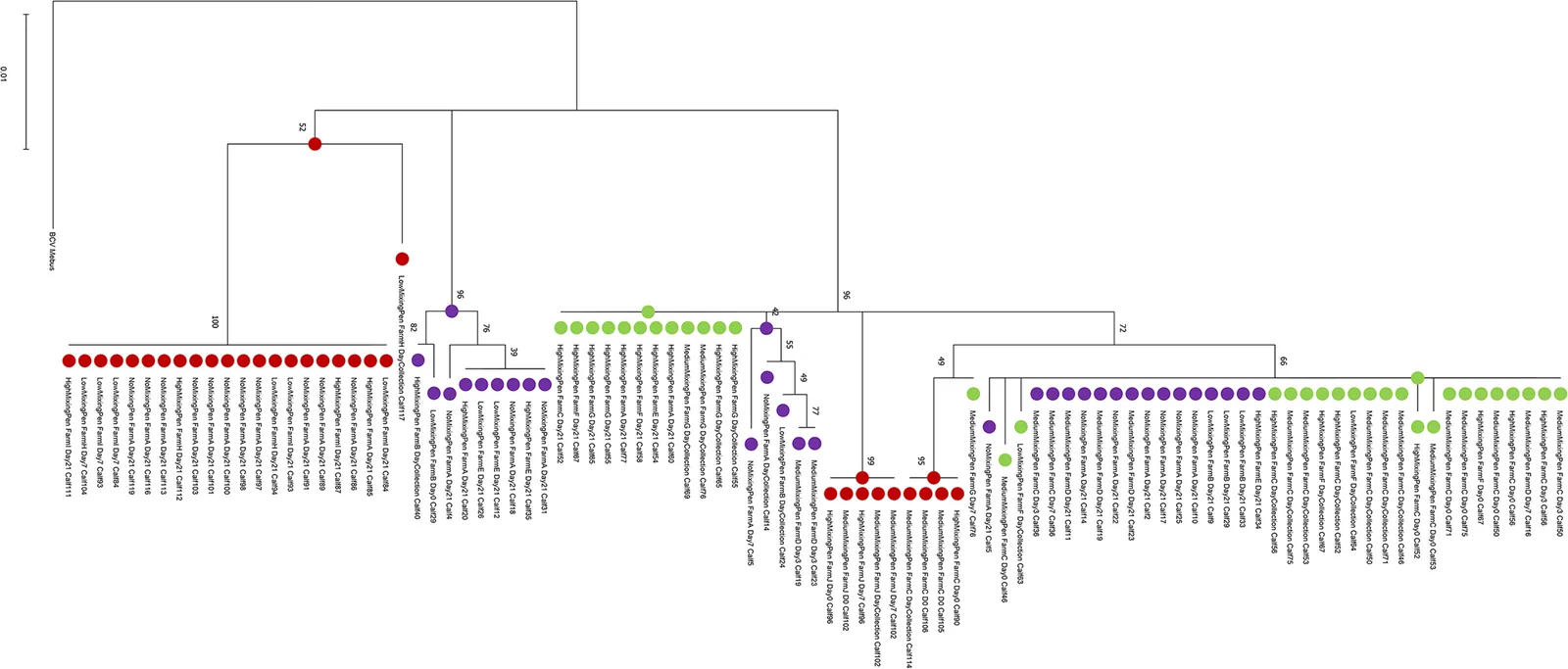
Phylogenetic tree from maximum likelihood analysis of bovine coronavirus spike protein hypervariable region (bp 1,368–1,776). Purple indicates cohort 1 isolates, green indicates cohort 2 isolates, and red indicates cohort 3 isolates. The reference Mebus strain (NCBI:txid11132) was included as the outgroup. The tree was bootstrapped at 1,000 replicates, and bootstrap values are shown.

One animal (calf 29) shed a different BCV genetic variant on d 21 postmixing compared with the mixing day (p-distance of ∼3.9%), whereas other isolates recovered from the same animal across different time points (e.g., calves 50, 53, 56, 84, 102) had HVR sequences that differed by ≤1 nucleotides, and belonged to the same terminal clade.

The BaTS analysis revealed a strong phylogenetic relatedness of sequences belonging to the same study cohort. Both AI and PS values were substantially lower than expected under the null model, based on trait randomization across the posterior distribution of trees (*P* < 0.001). Furthermore, group-specific MC analyses demonstrated that cohorts 1 and 3 showed particularly strong phylogenetic structure, forming larger monophyletic clades than expected by chance (*P* ≈ 0.01), while cohort 2 also showed significant but weaker clustering (*P* ≈ 0.04). Within each study cohort, sequences also clustered globally by farm of origin and study day, as assessed by AI (*P* < 0.01 across all evaluations). The PS and MC analyses were not interpreted for these traits due to small (n < 10) and uneven group sizes for certain farm and study day categories.

### Clinical Respiratory Disease

Variables affecting CPN (score ≥4 on clinical scoring system) during commingling were further evaluated using a GLMM.

After a priori inclusion of variables of interest (mixing treatment [pen type], mixing length, and nasal BCV shedding), and forcing-in control variable (study cohort), only the presence of fecal BCV shedding and CPN history during quarantine period remained in the final model as an additional meaningful fixed effect.

Mixing treatment (pen type; *P* = 0.033, ΔR^2^ = 0.09) had the largest effect on the odds of CPN. This was followed by mixing period (*P* = 0.060, ΔR^2^ = 0.050), fecal BCV shedding (*P* = 0.061, ΔR^2^ = 0.044), and CPN history during quarantine (*P* = 0.065, ΔR^2^ = 0.023) (Table 4). Specifically, calves in the medium and high-mixing pens had approximately 5-fold higher odds of CPN incidence than calves in the homogeneous (unmixed) pen (95% CI: 1.40–17.8 and 1.49–18.6, respectively). Furthermore, during the middle and late mixing periods (after the first week of mixing), calves had approximately 3-fold (1.07–8.47 and 1.14–9.91) higher odds of CPN compared with the first week (*P* = 0.035 and *P* = 0.028, respectively). Nasal BCV shedding within 7 d of symptom recording was not consistently associated with clinical disease symptoms.

**Table 4:** Factors associated with clinical pneumonia during a controlled mixing event, estimated using a generalized linear mixed model with a binomial distribution and logit link; results are presented as odds ratios (OR) with 95% CI^1^

Predictor	OR	95% CI (lower; upper)	*P*-value
Cohort			
Cohort 2	0.95	0.37; 2.46	0.92
Cohort 3	0.84	0.31; 2.20	0.71
Mixing period			
Middle (mixing d 7–14)	3.023	1.07; 8.47	0.04*
Late (mixing d 15–21)	3.34	1.14; 9.91	0.03*
Fecal BCV shedding	0.23	0.05; 1.07	0.06
Mixing treatment (pen type)			
High-mixing pen	5.00	1.40; 17.80	0.01*
Medium-mixing pen	5.26	1.49; 18.60	0.01*
Low-mixing pen	2.27	0.57; 9.03	0.24
Nasal BCV shedding			
Low shedding	1.36	0.57; 3.20	0.49
High shedding	1.71	0.38; 7.60	0.48
CPN history	2.61	0.94; 7.20	0.06

1 Reference categories were cohort 1, early period (d 2–6 after mixing), no nasal or fecal bovine coronavirus (BCV) shedding, homogeneous (no mixing) pen, and no clinical pneumonia during quarantine period. Low nasal BCV shedding was defined as <10^7^ copies/μL.

* *P* < 0.05.

### Lung Lesions

Variables occurring concurrently with LL were evaluated using a GLMM. After a priori inclusion of variables of interest (mixing treatment [pen type] and mixing length, and nasal BCV), and forcing-in control variable (study cohort), only the presence of fecal BCV shedding LL history on source farm remained as an additional meaningful fixed effect in the final model.

History of LL on source farm, (TUS ≥2 measured on source farm collection, 11–14 d before mixing), had the largest influence on the odds of LL during the mixing period (*P* = 0.0014, ΔR^2^ = 0.064). This was followed by study cohort (*P* = 0.014, ΔR^2^ = 0.060), fecal BCV shedding (*P* = 0.016, ΔR^2^ = 0.030), and length of mixing (*P* = 0.03, ΔR^2^ = 0.032; Table 5). Specifically, calves with a history of LL were approximately 4.65 times (95% CI: 1.81–11.93) more likely to have LL during the mixing period compared with calves without prior history. Furthermore, calves shedding fecal BCV were about 3 times more likely to have LL (1.14–17.4). Although mixing treatment (pen type; *P* = 0.167, ΔR^2^ = 0.033) did not significantly affect the odds of LL, the overall effect of nasal BCV shedding approached significance (*P* = 0.089, ΔR^2^ = 0.029). Notably, although low nasal shedding did not alter the odds of lesions, calves with high nasal viral shedding (≥10^7^ copies/µL) exhibited a 4.46-fold (1.21–6.8) increase in the odds of concurrent LL compared with nonshedders (*P* = 0.032).

**Table 5:** Factors associated with concurrent lung lesions (LL) during a controlled mixing event, estimated using a generalized linear mixed model (GLMM) with a binomial distribution and logit link; results are presented as odds ratios (OR) with 95% CI^1^

Predictor	OR	95% CI (lower; upper)	*P*-value
Cohort			
Cohort 2	0.25	0.01; 0.63	0.003**
Cohort 3	0.52	0.23; 1.18	0.12
Day 21 after mixing	0.46	0.23; 0.94	0.03*
Nasal BCV shedding			
Low shedding	1.08	0.48; 2.43	0.86
High shedding	4.46	1.21; 6.80	0.03*
Fecal BCV shedding	2.88	1.14; 17.4	0.02*
Mixing treatment (pen type)			
Low-mixing pen	2.96	1.06; 8.27	0.04*
Medium-mixing pen	1.39	0.50; 3.88	0.53
High-mixing pen	2.07	0.75; 5.67	0.16
LL history	4.65	1.81; 11.93	0.001**

1 Reference categories were cohort 1, mixing d 0, no nasal or fecal bovine coronavirus (BCV) shedding, homogeneous (no mixing) pen, and no LL history. Low nasal BCV shedding was defined as <10^7^ copies/μL.

* *P* < 0.05,

** *P* < 0.01.

Animals enrolled in cohort 2 had lower odds (OR = 0.25; 95% CI: 0.01–0.63) compared with cohort 1, and odds of having LL on d 21 after mixing was roughly half of the odds on the day of mixing (0.23–0.94).

### BCV Shedding and ADG

Variables affecting ADG during commingling were evaluated with a linear regression model. After a priori inclusion of variables of interest (mixing treatment [pen type], cumulative CPN/TUS scores, and nasal BCV shedding), and forcing-in control variable (study cohort), only ADG during quarantine period remained as an additional meaningful fixed effect in the final model. Descriptive statistics overview of growth characteristics of enrolled animals can be found in Supplemental Table S3 (see Notes).

Cohort (*P* < 0.001, ΔR^2^ = 0.13) and nasal BCV shedding (*P* = 0.004, ΔR^2^ = 0.07) had the biggest effect on ADG. Calves in cohort 3 gained approximately 0.4 kg/d less than calves in cohort 1 (95% CI: −0.59, −0.20), and calves shedding high nasal BCV (>10^7^ copies/µL) at any point during the mixing period gained on 0.38 kg/d less than nonshedders (−0.62, −0.14; Table 6). Mixing treatment approached statistical significance (*P* = 0.08), with calves in the high-mixing pen gaining ∼0.2 kg/d less than calves in the homogeneous pen (*P* = 0.07), as well as ADG during quarantine (*P* = 0.08), which was positively associated with ADG in the mixing period. Neither cumulative CPN score (*P* = 0.44), nor TUS category (*P* = 0.41) meaningfully affected ADG.

**Table 6:** Factors associated with ADG (kg/d) during a controlled mixing event, estimated using an ordinary least squares linear regression model; results are presented as regression estimates with 95% CI^1^

Predictor	Estimate	95% CI (lower; upper)	*P*-value
ADG during quarantine	0.12	−0.01; 0.25	0.07
Cohort			
Cohort 2	0.013	−0.17; 0.21	0.89
Cohort 3	−0.39	−0.59; −0.20	<0.001***
Nasal BCV shedding			
Low shedding	−0.021	−0.19; 0.14	0.80
High shedding	−0.38	−0.62; −0.14	0.002**
Cumulative clinical score	0.0027	−0.004; 0.01	0.44
Cumulative TUS score			
Moderate	0.084	−0.08; 0.25	0.32
Severe	0.13	−0.08; 0.34	0.22
Mixing treatment (pen type)			
Low-mixing pen	0.020	−0.20; 0.23	0.86
Medium-mixing pen	0.050	−0.17; 0.28	0.65
High-mixing pen	−0.19	−0.40; 0.01	0.07

1 Reference categories were cohort 1, no nasal or fecal bovine coronavirus (BCV) shedding at any point during the mixing period, homogeneous (no mixing) pen, and healthy thoracic ultrasonography (TUS) score. Cumulative TUS categories were defined as healthy (0–2), moderate (3–4), and severe (≥5) based on the cumulative score from 2 measurements at the beginning and end of the mixing period. Low nasal BCV shedding was defined as <10^7^ copies/μL.

** *P* < 0.01,

*** *P* < 0.001.

## DISCUSSION

### BCV Shedding During a Controlled Commingling Event

This study demonstrated that calves aged 10 to 12 wk have high levels of serum antibodies against BCV. Given the high prevalence of BCV on dairy farms (∼80% herd prevalence), it is reasonable to assume that the study calves had already been exposed to the virus (Berge and Vertenten, 2024). However, it should be noted that the ELISA assay used detects IgG_1_, which is also abundant in colostrum and has been shown to persist in the calf's circulation for up to 4 mo of age (Heath et al., 2012). Consequently, detected antibodies may have been partially of maternal origin, which would explain their slow decline over the study period.

Despite the presence of BCV serum IgG, viral shedding was not prevented, either nasally or in the feces. Consistent with previous reports, nasal and fecal shedding was strongly correlated (Cho et al., 2001; Hasoksuz et al., 2002), supporting the hypothesized dual tropism of BCV. The odds of BCV shedding increased after the first week of commingling; however, no meaningful association was observed between shedding and mixing treatment. This increase in shedding may be attributable to associated stress (e.g., unfamiliar pen-mates and increased competition), leading to immunosuppression or reactivation of latent infections (Niu et al., 2022), or to exposure to genetically distinct BCV variants. We have previously demonstrated that BCV RNA can be detected in airborne particles moving between pens (Urbaniec et al., 2025). If these particles contained infectious virions, they could have contributed to BCV transmission across pens, irrespective of assigned pen type.

### BCV Genetic Diversity

At present, there is no published information on genetic diversity of BCV strains circulating in the UK. However, analyses of BCV sequences from other countries indicate relatively low genetic diversity compared with many other RNA viruses, with isolates often sharing >98% sequence identities (Workman et al., 2017; Suzuki et al., 2020). These findings are consistent with our observations. Within each study cohort, BCV isolates had primarily identical HVR sequences within SF before mixing; however, they subsequently clustered into separate clades during the mixing period. Overall, this suggests simultaneous circulation of farm-specific BCV variants. Introduction of such variants into immunologically naïve populations, may contribute to commingling-associated BCV outbreaks. This interpretation is further supported by our detection of 2 BCV genetic variants in the same animal prior to and after mixing, and the observation that seropositivity did not prevent shedding. Serological cross-protection between BCV genetic variants is currently poorly understood; however, it has previously been shown to differ significantly between isolates from different phylogenetic clades (Fulton et al., 2013).

### BCV and BRD

Consistent with previous studies in calves of a similar age (Cho et al., 2001; Francoz et al., 2015), we did not observe a meaningful association between BCV shedding and clinical signs of respiratory disease. One possible explanation was the presence of high levels of circulating serum antibodies, which may have conferred protection against clinical disease without providing sterilizing immunity, therefore permitting continued viral shedding. Alternatively, BCV may primarily act as an opportunistic pathogen, as it has frequently been detected in samples collected from apparently healthy cattle (Fulton et al., 2011; Mitra et al., 2016). This hypothesis is further supported by mechanistic evidence, as BCV has been shown to increase the adherence of *Pasteurella multocida* to bovine respiratory epithelium in vitro (Fahkrajang et al., 2021). Finally, this study employed a modified Wisconsin–Madison clinical scoring system, which did not include induced cough as a scoring criterion. Induction of cough would have required daily handling of calves, which was avoided to minimize the risk of fomite transmission between pens. Induced cough has been identified as an important predictor in the Wisconsin–Madison system (Love et al., 2014); therefore, its exclusion may have reduced the sensitivity of the clinical scoring approach used in this study, despite lowering the positivity threshold.

Interestingly, although the mere presence of low-level nasal shedding was not associated with LL, calves with high nasal viral loads (>10^7^ copies/µL) or those shedding BCV in the feces had significantly higher odds of exhibiting LL (TUS score ≥2). This finding could support a gut-lung axis mechanism, where enteric infection contributes to systemic inflammation or immunosuppression, thereby impairing the resolution of pulmonary lesions. Our observation that these associations persisted even in calves with high-titer maternal antibodies further suggests that circulating IgG_1_ is insufficient to mitigate the systemic impacts of BCV infection during commingling.

Furthermore, we report a clear association between both the duration and intensity of mixing and the odds of animals developing clinical BRD, with a significant increase in incidence after the first week of mixing and with greater mixing intensity. Although exposure to stressors such as commingling is often unavoidable in modern commercial production systems, efforts to minimize both the duration and severity of these exposures where feasible, for example by limiting the extent of mixing at auction markets, are likely to reduce the risk of commingling-associated BRD outbreaks.

### BCV Shedding and Growth Rate

We observed a strong association between high nasal BCV shedding and reduced average daily gain. Although this relationship has not been extensively studied, Lathrop et al. (2000) reported no association between BCV shedding and ADG which contrasts with our findings. However, the aforementioned study did not evaluate shedding intensity. In our analysis, we also found no association between ADG and low-level shedding, which suggest a potential functional distinction between active infection (high shedders) and transient low-level viral shedding. Supporting this interpretation, Hasoksuz et al. (2002) reported that BCV seroconversion was inversely associated with ADG, even in the absence of clinical signs. Bovine coronavirus infection could potentially negatively affect growth performance by inducing inflammatory pathways, reducing feed conversion efficiency, or altering endocrine responses such as increased cortisol levels, even when overt clinical signs are not present (Niu et al., 2022).

### Limitations

This study was subject to several limitations. First, practical constraints on the number of available calves from each SF meant that some SF contributed to only one cohort, whereas the homogenized (control) group originated from the same SF across all 3 cohorts. This may have introduced confounding; however, we attempted to account for this by including cohort as a fixed effect in all models.

Second, although calves within each mixing group were housed in separate pens with no possibility of direct contact, the outdoor housing system meant that indirect transmission via aerosols could not be completely excluded and would have depended on environmental conditions (i.e., wind, temperature, humidity). Although BCV is considered to be transmitted primarily via direct contact (Saif, 2010), BCV RNA was detected in air samples between pens. This is described in more detail in a companion study (Urbaniec et al., 2025). However, even if airborne transmission between pens did occur, it is unlikely to have materially affected the conclusions presented in this study.

Furthermore, as calves were sold to commercial farms at the end of the study period, long-term follow-up was not possible. Given that both BCV shedding and respiratory disease were most prevalent at later time points, potentially important longer-term associations may have been missed.

Finally, the premixing quarantine period differs from standard commercial calf-raising practices, which may limit the generalizability of reported BCV transmission dynamics.

## CONCLUSIONS

Overall, our results suggest that during commingling BCV circulates widely as a predominantly subclinical infection, and is associated with negative growth outcomes. Conversely, the development of clinical respiratory disease is more strongly influenced by the duration and intensity of commingling rather than by BCV shedding alone. This highlights the importance of on-farm management strategies aimed at reducing respiratory pathogen burden to maximize efficiency and production, not simply treatment of overt respiratory disease.
